# Tuning all-Optical Analog to Electromagnetically Induced Transparency in nanobeam cavities using nanoelectromechanical system

**DOI:** 10.1038/srep14379

**Published:** 2015-09-29

**Authors:** Peng Shi, Guangya Zhou, Jie deng, Feng Tian, Fook Siong Chau

**Affiliations:** 1 Department of Mechanical Engineering, National University of Singapore, 9 Engineering Drive 1, Singapore 117576; 2 Institute of Materials Research and Engineering, A*STAR (Agency for Science, Technology and Research), 3 Research Link, Singapore 117602

## Abstract

We report the observations of all-optical electromagnetically induced transparency in nanostructures using waveguide side-coupled with photonic crystal nanobeam cavities, which has measured linewidths much narrower than individual resonances. The quality factor of transparency resonance can be 30 times larger than those of measured individual resonances. When the gap between cavity and waveguide is reduced to 10 nm, the bandwidth of destructive interference region can reach 10 nm while the width of transparency resonance is 0.3 nm. Subsequently, a comb-drive actuator is introduced to tune the line shape of the transparency resonance. The width of the peak is reduced to 15 pm and the resulting quality factor exceeds 10^5^.

The one-dimensional (1D) photonic crystal (PC) nanobeam cavity has attracted much attention lately because of its merits, such as small dimensions, ease of fabrication and better integration with optical waveguides^1^. Recently, more advanced PC nanobeam cavities with ultrahigh quality factor (Q-factor) have been reported^2^,^3^,^4^,^5^. Among these, the “zipper” cavity can simultaneously localize mechanical and optical energy at the nanoscale^6^. In addition, a deterministic design method for cavities with ultrahigh Q-factor and ultrahigh transmission has been proposed and experimentally demonstrated. This design method is ideally suited for coupling to the feeding waveguide due to its ultrahigh transmission. Moreover, the cavity’s parameters can be determined directly, hence avoiding trial-based parameter-search simulations. Furthermore, the resonances of coupled nanobeam cavities can be tuned by on-chip-integrated nanoelectromechanical systems (NEMS)-based actuators^7^,^8^.

Quantum coherence in atomic systems can lead to fascinating and counter-intuitive phenomena, such as laser cooling, trapping of atoms, and Bose-Einstein condensates. Electromagnetically induced transparency (EIT) in atomic vapors has attracted much research interest in recent years. Due to the quantum destructive interference between excitation pathways to the upper level in three-level atomic systems, EIT leads to a sharp cancellation of absorption in the medium^9^,^10^, resulting in phenomena such as lasing without inversion, freezing light^11^, and dynamical storage of light in a solid-state system^12^, which can be used for efficient quantum entanglement generation and nonlinear optical processes^13^,^14^,^15^. However, the narrowness of the EIT window and the complexity of constructing atomic vapor systems restrict the practical use of the EIT effect. Similar to atomic systems, where interference is driven by a coherent laser field, EIT-like effects can be observed through classical means^16^. Several theoretical proposals have suggested this intriguing possibility in an optical analogy to atomic EIT^17^,^18^,^19^,^20^,^21^,^22^,^23^,^24^, overcoming much of the limitations on decoherence and bandwidth from electronic states for EIT, with applications in stopping and trapping light at room temperature. Furthermore, classical all-optical analogies are also realized on various platforms, including PC waveguides and micro-cavities^25^,^26^, plasmonic nanostructures, and metamaterials^27^,^28^. Slow light in photonic structures has been examined but is likewise bounded by the fundamental limit of delay bandwidth. Recently, EIT-like effects were examined experimentally in two coupled whispering-gallery mode resonators^29^,^30^,^31^, which can store light on-chip beyond the static bandwidth limit. Particularly, coherent interference between resonant modes induces the absence of absorption in coupled optical resonators.

In this paper, we first describe the design of PC nanobeam cavities to achieve EIT. Through changing the gap between side-coupled waveguide and cavities, the phenomenon of all-optical EIT in multiple standing-wave PC nanobeam cavities, caused by the Fabry–Pérot interference of two cavities modes, can be experimental observed. Finally, we experimentally demonstrate the resonance tuning of EIT resonance by means of an on-chip integrated NEMS actuator.

## Design

The design of the nanobeam cavities is based on Silicon-on-Insulator (SOI) wafers with a device layer of 260 nm and buried oxide of 2 μm. This SOI-based design is desirable for future large-scale integration of photonic and electronic devices. The PC geometry of air-holes in dielectric is favorable for transverse-electric-like (TE-like) band gaps. Hence in this paper, we study TE-like modes. The resonance of a PC cavity is formed by introducing a “defect” into the photonic lattice. These defect-type cavities allow wavelength-scale localization with ultrahigh Q-factors^32^ for nonlinear^33^ and quantum optics^34^. There are many design methods for various ultrahigh Q-factor 1D PC nanobeam cavities, including lattice-constant-modulation, hole-size-modulation and beam-width-modulation^3^,^4^,^5^. We adopt the hole-size-modulation method here. We utilize 41 holes to constitute the nanobeam cavity and set all these holes as the “defect” region. The lattice period of the cavity *p* is fixed at 360 nm. The radius of the air-holes is labeled by *r*_*n*_, (*n* changes from 0 to 20, corresponding to the number of holes from the center to either side in the cavity region). The radius in the “defect” region is modulated according to the equation:


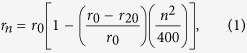


where *r*_*20*_ = 60 nm and *r*_*0*_ = 150 nm, respectively. In addition, the width of cavity is *w* = 680 nm. With the refractive index of silicon set to 3.476, the three-dimensional (3D) finite-difference time-domain (FDTD) simulations for these structures are carried out using RSoft FullWAVE.

**Figure 1:**
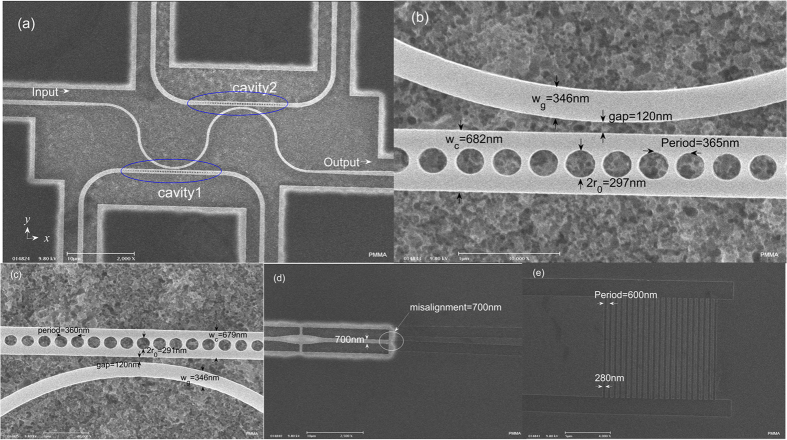
(**a**) Global SEM image of the nanostructure to realize all-optical analog of EIT. The light grey area shows the released region and the device layer undercut is about 1.2 μm. (**b**) The magnified SEM image and detailed dimensions for fabricated cavity1. (**c**) The magnified SEM image and detailed dimensions for fabricated cavity2. (**d**) Joint of rib waveguide and air-suspended waveguide. A misalignment is clearly seen. (**e**) Grating coupler designed for coupling light between chip under test and fibers.

The system as shown in Fig. 1(a) consists of a silicon waveguide side-coupled to two high intrinsic Q-factor PC nanobeam cavities. The oxide layer under the cavities and nanobeam waveguide has been removed at the end of the fabrication process, thereby creating released air-suspended nanostructures. All the dimensions except for the nanobeam thickness (*t* = 260 nm) of the fabricated device are annotated in Fig. 1.

The structure presented here to measure EIT-like line shapes can be analyzed with the coupled-mode formalism^26^,^35^. The theoretical model can be considered as two cavities modes connecting with each other through a waveguide. The phase change caused by the round trip in the waveguide determines the line shape of the resulting EIT-like peak. The dynamical equations for the two cavity mode amplitudes are^35^:


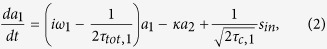



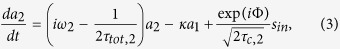


where *a*_1_ (*a*_2_) is the cavity mode amplitude in cavity1 (cavity2), *s*_*in*_ indicates the incident guided-mode field, *ω*_1_ and *ω*_2_ denote respectively the resonance frequencies of cavity1 and cavity2, 1/*τ*_tot,1_ (1/*τ*_tot,2_) is the total decay rate of the cavity1 (cavity2), and 1/*τ*_c,1_ (1/*τ*_c,2_) is the decay rate due to the waveguide coupling loss of the cavity1 (cavity2). Additionally, 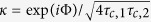, where Φ = *ωn*_*eff*_*L*/*c* is the phase difference between cavities with *ω* being the incident guided-mode frequency and *c* being velocity of light in vacuum. The waveguide mode index *n*_*eff*_ is 1.9996 at wavelength 1.57 μm for the designed waveguide of length L ≈ 31.42 μm and width of 360 nm. Then, we can calculate the phase difference between two cavities as being 40*2π, which makes the cavities modes capable of constructing Fabry–Pérot interference. However, there will be inevitable deviations in the nanofabrication process. Thus the EIT-like resonance would be slightly asymmetric when the round-trip phase difference 2Φ is not an integer multiple of 2π. The total loss rate 1/τ_tot_ = 1/τ_c_ + 1/τ_int_, where τ_c_ = Q_c_/ω and τ_int_ = Q_int_/ω are the cavity decay constants into the side-coupled waveguide and free space (intrinsic loss), respectively. The details of the coupled mode theory with PC microcavities are presented in refs 36, 37, 38.

## Experimental Results

**Figure 2:**
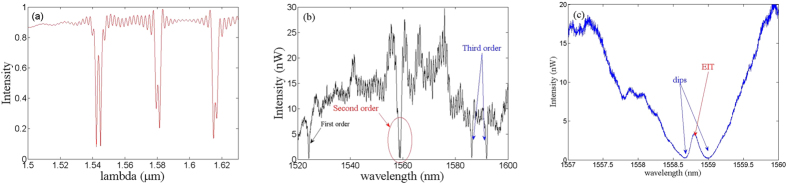
(**a**) The simulated spectrum of the structure consisting of a waveguide side-coupled to two cavities with periods equal to 360 nm and 360.5 nm, respectively. The calculated phase differences Φ are 41.9 × 2π and 39.4 × 2π, respectively for the lowest two modes. (**b**) The measured spectrum for the structure shown in Fig. 1(a). There are five dips in the spectrum due to the side-coupled cavities. The modes are labeled in the figure. (**c**) The magnified spectrum for the interference of the second modes. A peak is present owing to the coherent interference and the resonance wavelength of EIT-like peak is at 1558.8 nm.

From the global SEM image of the fabricated device, it is seen that there is a silicon waveguide side-coupled to two high intrinsic Q-factor PC nanobeam cavities. For designed cavity1 with the period of PC cavity set to 360 nm, the calculated wavelength and intrinsic Q-factor (Q_int_) for the fundamental mode are respectively 1540.37 nm and 1.4 × 10^8^, while those of the second mode are 1578.21 nm and 5.6 × 10^6^, respectively. However, for designed cavity2, the period is slightly changed to 360.5 nm. The corresponding resonance wavelengths (Q-factors) of the fundamental and second modes are 1540.38 nm (3.6 × 10^7^) and 1578.22 nm (5.6 × 10^6^), respectively. The differences are not obvious due to the extremely small change of period. The simulated spectrum is shown in Fig. 2(a), from which it is obvious that coherent interferences are present. The modal effective indices of the first two dips are calculated to be about 2.055 (1543.6 nm) and 1.982 (1580 nm), respectively. The calculated phase differences Φ are 41.9 × 2π and 39.4 × 2π, respectively. It can also be observed that the interference line shape of fundamental modes is almost symmetric, while that of the second and third modes are asymmetric. The measured spectrum is given in Fig. 2(b). The high level of noise results from the misalignment of the air-suspended and rib waveguides. It is obvious that there are five dips in the experimental spectrum. Since the fabricated period of the cavity1 (365 nm) is larger than that of cavity2 (360 nm), the first order mode for cavity1 is beyond the measurement region. As we carefully observe the measured spectrum, the second order resonance of the two cavities is coherent. There is a peak in the intersection of two dips, which can be considered as coherent interference, which is similar with the coherent interference in EIT. The measured Q-factor (Q_tot_) for the fundamental mode is 1840, which means that the waveguide-cavity coupling Q-factor (Q_c_) is about 1840. It is obvious that the cavity is operated in the strongly over-coupled regime because the measured Q_int_/Q_c_ is around 10^5^. From the ref. 22, the resonances λ_1_ (1558.67 nm) and λ_2_ (1559 nm) of two cavity are overlapping, and δ = 2τ_tot_(c/λ_1_–c/λ_2_) ≠ 0, where τ_tot_ total is the loaded life-time of a single cavity. Moreover, since the round-trip phase 2Φ between the two cavities satisfies the condition of a Fabry-Perot resonance, the system can therefore represent an all-optical analogue of EIT with two degenerate modes at (λ_1_ + λ_2_)/2. From coupled-mode theory analysis^35^, as δ < 3.5, the condition of EIT-like coherent interference is satisfied and we can get Q_EIT_ > Q_tot_. The lifetime of these two degenerate modes with EIT-like spectral features will decay more slowly than that of a single cavity. From Fig. 2(c), the experimental detuning is obtained to be around δ = 0.779 and the EIT-like resonance can be seen in the results. Moreover, the calculated phase difference Φ equals 39 × 2π, which makes the EIT-like resonance symmetric. Using the Lorentz fit, the experimental Q_EIT_ for the EIT-like peak is 17600, which is about 10 times that of a single cavity.

**Figure 3:**
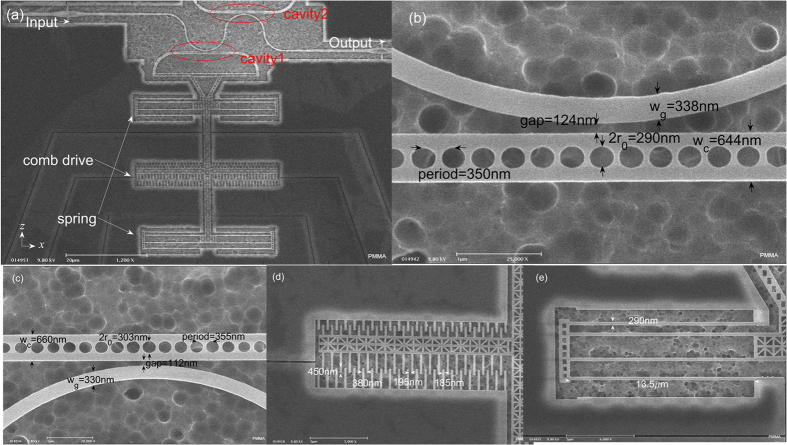
(**a**) Global SEM image of electrically tunable waveguide-side-coupled nanobeam cavities with NEMS comb drive actuators. The light grey outline shows the released region with undercut about 1 μm, which means that the comb-drive can be moved in the z-direction. (**b**) The magnified SEM image and detailed dimensions for the fabricated cavity1. (**c**) The magnified SEM image and detailed dimensions for fabricated cavity2. (**d**) The magnified SEM image for actuator’s critical dimensions. The width of comb-drive fingers is 195 nm; initial finger overlap is 450 nm; the air gap between two adjacent fingers is 185 nm; the finger number is 41 on each side. (**e**) The magnified SEM image of a NEMS spring. The width and length of the flexible beams are 290 nm and 13.5 μm respectively.

Subsequently, we fabricate a structure with a waveguide side-coupled to two cavities and an integrated NEMS comb-drive actuator to investigate the coupling effect between cavities and waveguide, as shown in Fig. 3(a). The electrostatic pulling force is generated by the potential difference between the two sets of comb-drive fingers. Normally, the movable part of the comb-drive is connected with the ground to avoid its bending towards the grounded substrate while a positive voltage is applied to the fixed electrode of the comb-drive. Cavity1 labeled in Fig. 3(b) is connected with the movable part of the comb-drive, while the cavity2 is fixed for calibration purposes. The current design allows bi-directional movement and thus bipolar tuning is feasible experimentally. The gap between cavity1 and waveguide can be widened or narrowed when a voltage (V_a_) is applied to actuate the downside or upside of the comb-drive. In the presented device, the width of the comb drive fingers, initial finger overlap and the air gap between two adjacent fingers are given in Fig. 3(d). For clarity, one of the springs in the NEMS actuator and its dimensions are shown in Fig. 3(e).

The relationship between displacement and applied voltage can be approximately calculated by^39^:


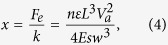


where *F*_*e*_ is the electrostatic force produced by the comb-drive actuator, *k* is the spring constant of the folded beams, *n* is the number of comb-drive fingers, *ε* is the permittivity in air, *t* is the device thickness, *V*_*a*_ is the applied voltage, *s* is the air gap between two adjacent fingers, *E* is the Young’s modulus of the beam material, and *w* and *L* are the width and length of the folded beams, respectively. Using Eq. (4), the movement *x* is about 30 nm when the applied voltage equals 10 V and around 60 nm as the applied voltage is increased to 15 V.

**Figure 4:**
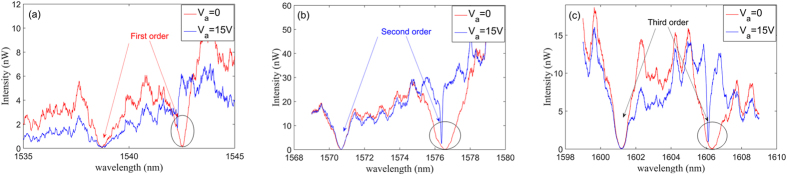
The measured spectrum of (a) fundamental, (b) second and (c) third modes of the nanostructure shown in **Fig. 3(a)**. The red lines show the transmission spectrum when the applied voltage is off, while the blue lines show the measured spectrum as the voltage is raised to 15 V.

The measured spectra of the structure in Fig. 3(a) are shown in Fig. 4. The red lines give the original spectrum when the applied voltage is off. There are two dips in each figure, of which the left one is the resonance of cavity2. The left dips are almost unchanged as the voltage is applied and are just introduced for calibration purposes. The right dips give the resonances of cavity1. The experimental spectrum undergoes an obvious change when the voltage is applied. The gap between cavity1 and waveguide is increased to 180 nm when the applied voltage is 15 V. Furthermore, the second and third modes are almost critically coupled with the waveguide and the resulting Q-factors are more than 10000.

**Figure 5:**
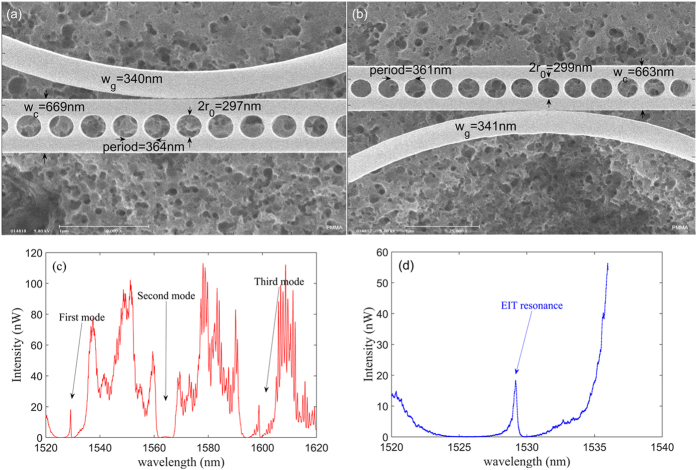
(**a**) The magnified SEM image and detailed dimensions for fabricated cavity1. (**b**) The magnified SEM image and detailed dimensions for fabricated cavity2. The pattern is similar with Fig. 1(a) except for the smaller gap between cavities and waveguide, which leads to larger overcoupling (Q_int_/Q_c_ ≈ 10^6^). (**c**) The measured spectrum for the nanostructure with a 10 nm gap between waveguide and the cavities. EIT-like phenomenon can be clearly seen. (**d**) The magnified spectrum for the interference of the fundamental modes. An EIT peak is present owing to the coherent interference and the peak is at 1529.14 nm.

We surmise that decreasing of the gap would further increase the over-coupling effect and make Q_c_ lower. Arising from this analysis, we fabricate a structure with the gap between the cavities and waveguide exceedingly small (≈10 nm), as shown in Fig. 5(a,b). The resonance of the cavity can be greatly affected for the near-field coupling of cavity. The experimental Q_c_ of the cavity is downgraded to 200 as seen from the experimental spectrum in Fig. 5(c). Thus, the measured Q_int_/Q_c_ of the fundamental mode is around 10^6^, and the Q factor for the EIT-like resonance is Q_EIT_ = 5000, which is 30 times that of a single cavity. The measured width of the waveguide is 340 nm and the effective index equals 1.9618. From the coupled mode theory, the phase difference Φ and cavity-cavity detuning δ for the fundamental modes are 40.3 × 2π and 2.82, respectively, which can be deduced from the asymmetric line shape and obvious EIT-like resonance seen in Fig. 5(d). Since we can obtain the detuning δ = 3.6 > 3.5 when the Q_tot_ is set to 200 by carefully checking the details of spectrum, we note that the EIT behavior disappears for the second modes. Additionally, the bandwidth of the fundamental mode can extend 8 nm, while that of third mode is around 10 nm, which are much broader than those reported in ref. 26.

**Figure 6:**
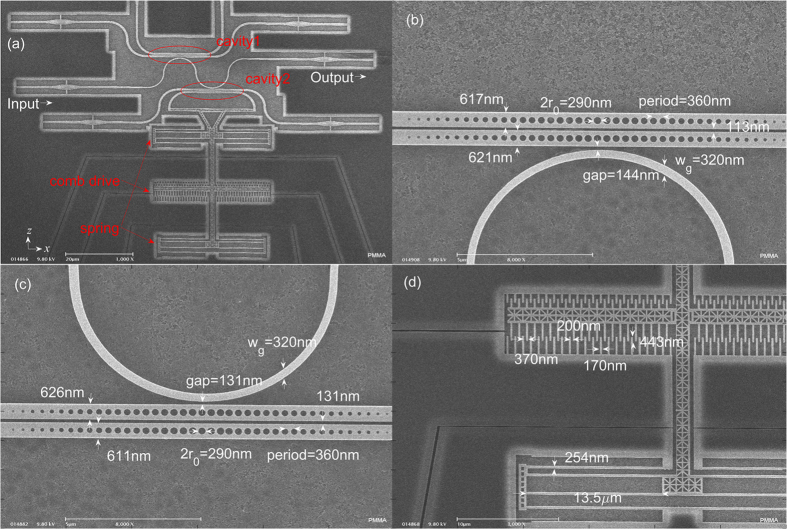
(**a**) Global SEM image of electrically tunable coupled nanobeam cavities side-coupled to a waveguide and integrated with a NEMS comb drive actuator. The light grey outline shows the released region with undercut about 1.5 μm, which means that the comb-drive is movable in the z direction. (**b**) The magnified SEM image and detailed dimensions for fabricated coupled cavity1; (**c**) The magnified SEM image and detailed dimensions for fabricated coupled cavity2. (**d**) The magnified SEM image showing the actuator’s critical dimensions. The width of comb-drive fingers is 170 nm; initial finger overlap is 443 nm; the air gap between two adjacent fingers is 200 nm; the finger number is 41 on the each side. The width and length of the flexible beams in NEMS springs are 254 nm and 13.5 μm respectively.

**Figure 7:**
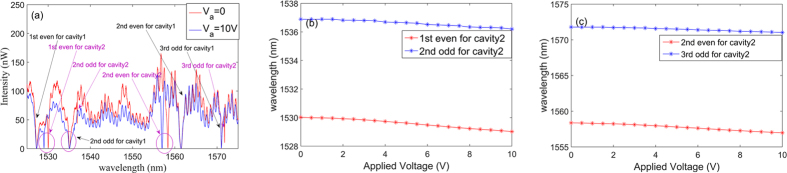
(**a**) The measured spectrum of the nanostructure shown in Fig. 6(a). The red lines show the transmission spectrum when the applied voltage is at 0 V, while the blue lines show the measured spectrum at 10 V. (**b**) The dependence of experimental resonance on the applied voltage with increments of 0.5 V. The red and blue lines show the fundamental even and second odd modes, respectively. (**c**) The dependence of experimental resonance on the applied voltage with increments of 0.5 V. The red and blue lines show the second-order even and third odd modes, respectively.

Finally, we fabricate a nanostructure with a waveguide side-coupled to two coupled nanobeam cavities, as shown in Fig. 6(a). The detailed dimensions of coupled cavity1, coupled cavity2 and NEMS actuator are given in Fig. 6(b–d), respectively. The designed dimensions of the coupled nanobeam cavities are identical to those of the proposed structure in Fig. 1(b) except that the gap between coupled cavities is designed to be 100 nm. The simulated wavelengths (Q factors) for the first even, second odd, second even and third odd modes are 1539.4 nm (7.9 × 10^6^), 1548.8 nm (7.4 × 10^6^), 1577.1 nm (2.0 × 10^6^) and 1588.3 nm (2.2 × 10^6^), respectively. The measured spectra with the applied voltage turned off and on are respectively shown in the red and blue lines of Fig. 7(a). Figure 7(b,c) show the dependence of resonance wavelengths of the fundamental, second, and third-order modes on the applied voltage with the increment set to 0.5 V. The blue and red lines show the odd and even modes, respectively. At an applied voltage of 10 V, the gap between the coupled cavities is increased to about 160 nm. From classical electromagnetic theory, the modal frequency goes up when the gap between two cavities scales up. These experimental results are consistent with those reported in ref. 8. However, the change of wavelength for each mode is about 1 nm. Although the tuning region of resonance in the structure is not very large, it is sufficient for tuning the EIT-like peak from the two third-order odd modes.

**Figure 8:**
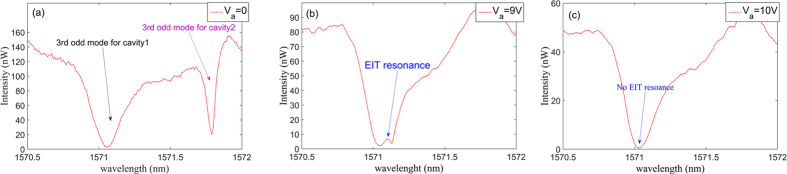
The detailed coherent interference spectrum of the third-order odd resonances for the nanostructure shown in Fig. 7(a). (**a**–**c**) show the spectra at the applied voltages of 0 V, 9 V and 10 V, respectively. The interference intensifies and the EIT-like peak is observed at the applied voltage of 9 V. The wavelength of the EIT-like peak is located at 1571.1 nm, and second the full width at half maximum (FWHM) is about 30 pm. At the applied voltage of 10 V, the two resonances overlap, and third the EIT-like phenomenon disappears.

The detailed coherent interference spectra of the third-order odd modes are show in Fig. 8(a–c) at the applied voltages of 0, 9 V and 10 V, respectively. The interference of the two modes is weak when the voltage is off. Since the coupled cavity1 and its coupling gap with the waveguide are unchanged as the voltage is applied, the measured resonance wavelength and Q-factor of the third-order odd mode for cavity1 are fixed at 1571.06 nm and 4500, respectively. When the voltage applied to the coupled cavity2 is at 0 V, the resonance wavelength and Q-factor of the third-order odd mode are 1571.79 nm and 30000, respectively. Moreover, when the applied voltage is 9 V, the interference intensifies and the EIT-like peak is observed. The wavelength of EIT-like peak is located at 1571.1 nm, and the experimental Q-factor Q_EIT_ is about 50000. The asymmetric line shape is due to Φ ≠ 2nπ. The measured Q_EIT_ factor exceeds 10^5^ at an applied voltage of 9.5 V. From Fig. 8(c), it is seen that the two resonances overlap when the voltage is 10 V and the EIT-like phenomenon disappears. This is in accordance with the results theoretically predicted above.

**Figure 9:**
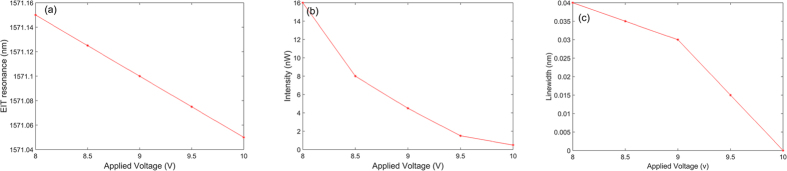
(**a**) The peak wavelength of the EIT resonance versus the applied voltage; (**b**) the transmitted intensity of the EIT resonance versus the applied voltage; (**c**) the linewidth of the EIT resonance varies as a function of the applied voltage.

We then summarize the measured peak wavelength, transmitted intensity, and linewidth of the EIT resonance as functions of the applied voltage. The results are shown in Fig. 9. Since the interference between the two cavity resonances is very weak when the applied voltage is smaller than 8 V, we only show the results from 8 V to 10 V with an increment of 0.5 V. It is observed that the peak wavelength, transmitted intensity, and linewidth of EIT resonance vary monotonously with the applied voltage. Furthermore, when the applied voltage is 10 V, the EIT resonance disappear and the linewidth of EIT resonance is 0, which agrees with the results shown in Fig. 8(c).

## Discussion and Conclusion

For cavity resonators, the total Q-factor (*Q*_*tot*_) can be estimated from the Q-factors of intrinsic loss (*Q*_*int*_) and waveguide coupling loss (*Q*_*c*_) using the following relation: 1/*Q*_*int*_ + 1/*Q*_*c*_ = 1/*Q*_*tot*_. If the cavity is critically coupled to the waveguide, then *Q*_*c*_ = *Q*_*int*_, and subsequently *Q*_*tot*_ = *Q*_*int*_/2. However, when the cavity and waveguide are over-coupled (*Q*_*int*_ > *Q*_*c*_) like the cases we investigated in this paper, the resonance mode decays much faster into the waveguide than into the air, and thus the *Q*_*tot*_ depends mainly on the *Q*_*c*_. In our design here, the *Q*_*int*_ of single cavities is larger than that of the coupled cavities. To investigate the *Q*_*c*_, we consider a fixed coupling gap for the cavity-waveguide coupling. By checking the modal profiles of a single cavity and coupled cavities, we find that the fraction of the optical field energy inside the waveguide is larger for the single cavity than that for the coupled cavities. This is due to the fact that modal volume of the single cavity is much smaller than that of the coupled cavities. Consequently, for identical coupling gaps, the coupling loss of the single cavity to the waveguide is larger thus resulting in a lower *Q*_*c*_. This may explain why the measured Q-factors of optical modes of coupled cavities are generally larger than those of single cavities, although the designed intrinsic Q-factor *Q*_*int*_ is higher in single cavities. The over-coupling in single cavities is accordingly larger, which makes *Q*_*int*_/*Q*_*c*_ (10^5^) of single cavities several orders of magnitude higher than that of couple cavities (10^2^). Therefore, the EIT-like linewidth of coherent interference is much narrower for coupled cavities. However, since there will generally be errors caused by the nanofabrication process, the tunability and applicability of coupled cavities are more flexible than those of single cavities, and hence the coupled cavities may be used to produce the multiple EIT-like phenomenon.

In summary, several structures are designed and fabricated to experimentally demonstrate an all-optical analog to EIT. With proper design of the two PC cavities with wavelength-scale localization, the distinctive EIT-like line shapes are observed, with measured EIT-like linewidths much narrower than for individual resonances. The Q-factor of EIT can be 30 times larger than that of measured individual resonances. Furthermore, when the gap between cavity and waveguide is reduced to 10 nm, the bandwidth of the destructive interference region can reach 10 nm while the FWHM of the EIT-like resonance is 0.3 nm. Subsequently, when a NEMS actuator is introduced to tune the line shape of resonance and EIT-like peaks, the FWHM of peak is reduced to 15 pm and the resulting Q-factor is 10^5^. The structure can be utilized to achieve multi-level coherently-coupled cavities. The experimental results support efforts toward realization of photon pulse trapping, dynamic bandwidth compression, and coupled-cavity quantum electrodynamics in scalable PC cavity arrays. Furthermore, the proposed structures could also be applied to gas sensing.

## Method

### Fabrication process

The device is fabricated on a silicon-on-insulator (SOI) wafer. E-beam resist such as ZEP 520A-7 is coated on the SOI wafer, forming a 260 to 270 nm-thick layer. The patterns of the nanostructures are obtained by electron beam lithography (EBL). The writing current is 200 pA and the exposure dose is 320 μC/cm^2^. The device pattern is transferred to the wafer’s device layer through an inductively-coupled plasma reactive ion etching (ICP-RIE) system, which uses plasma of C_4_F_8_/SF_6_ gas. The silicon layer is etched through in this step. The residual E-beam photoresist is removed by microposit 1165 remover. Another EBL and ICP-RIE etch are used to fabricate rib waveguides and grating couplers, except that the etching depth in this step is controlled to be at 80 nm. The residual E-beam photoresist is also removed by 1165. In addition, the other structures for characterizing the device, including the isolation trench, electrodes, etc, are fabricated through a series of lithography, RIE etching, metal E-beam evaporating, and lift-off processes. Finally, the wafer is diced into 6 × 6 mm chips, and the chips are then suspended on hydrofluoric (HF) acid vapor to etch the silicon dioxide below the cavities and actuators. One of fabricated structure is shown in Fig. 1, where 1(a) shows the global structure of the proposed waveguide side-coupled to two nanobeam cavities. The detailed images of cavity1 and cavity2 are shown in Fig. 1(b,c), respectively. Figure 1(e) is the SEM image of the grating coupler, whose area, period, filling factor (groove fraction of grating period) and groove depth are 12 μm × 12 μm, 600 nm, 46.7% and 80 nm respectively. This grating coupler has an experimentally-measured maximum of 30% coupling efficiency at a wavelength of ~1570 nm and a 3 dB-bandwidth of 105 nm. The light launched from the grating coupler is guided by a rib waveguide, which has the same etched depth (80 nm) as the grating coupler. The width of the rib waveguide then gradually tapers down to 700 nm, where it is connected to the air-suspended silicon waveguide as shown in Fig. 1(d). The misalignment reaches 700 nm caused by unknown issues during the two steps of EBL. This misalignment would result in reduction of transmission and increase in measurement noise.

### Measurement setup

Light from a tunable laser source (ANDO AQ4321D) is launched into a single-mode fiber. A fiber polarization controller is utilized to selectively excite the TE-like modes of the cavities. A pair of XYZ-stages control the coupling fibers to tilt 10 degrees from the normal direction to the device surface. The fibers are aligned manually under an optical microscope with the grating couplers on the device. Light coupled into the waveguide through the input grating coupler is directed to the cavity and the cavity’s output is launched into a multi-mode fiber through the output grating coupler. Finally, the light signal is recorded by an optical spectrum analyzer (ANDO AQ6317C). The TLS and the OSA can synchronously sweep through wavelengths from 1520 nm to 1620 nm with a resolution of 1 pm.

## Additional Information

**How to cite this article**: Shi, P. *et al.* Tuning all-Optical Analog to Electromagnetically Induced Transparency in nanobeam cavities using nanoelectromechanical system. *Sci. Rep.* **5**, 14379; doi: 10.1038/srep14379 (2015).
